# Factors associated with elevated low-density lipoprotein cholesterol levels among hill tribe people aged 30 years and over in Thailand: a cross-sectional study

**DOI:** 10.1186/s12889-021-10577-3

**Published:** 2021-03-12

**Authors:** Niwed Kullawong, Tawatchai Apidechkul, Panupong Upala, Ratipark Tamornpark, Vivat Keawdounglek, Chanyanut Wongfu, Fartima Yeemard, Siriyaporn Khunthason, Chalitar Chomchoei

**Affiliations:** 1 School of Health Science, Mae Fah Luang University, Chiang Rai, Thailand; 2 Center of Excellence for The Hill tribe Health Research, Mae Fah Luang University, Chiang Rai, Thailand; 3 Chulabhorn Royal Academy, Bangkok, Thailand

**Keywords:** Low-density lipoprotein cholesterol, Hill tribe, Prevalence, Factors associated

## Abstract

**Background:**

Low-density lipoprotein cholesterol (LDL-C) is one of the most important types of cholesterol and has an impact on health. Certain lifestyle and dietary habits in different populations may leads to increased levels of LDL-C, particularly among those with poor education and economic statuses, such as hill tribe people in Thailand. This study aimed to estimate the prevalence of and determine the factors associated with high LDL-C levels among hill tribe people in northern Thailand.

**Methods:**

A cross-sectional study was performed to gather information from six main hill tribe populations: Akha, Lahu, Hmong, Yao, Karen, and Lisu. Individuals who were aged over 30 years and living in 30 selected hill tribe villages were invited to participate in the study. A validated questionnaire and 5-mL blood specimens were used to obtain data. Correlation analyses, chi-square tests, t-tests, and logistic regression were used to detect correlations and associations.

**Results:**

A total of 2552 participants were recruited into the study; 65.9% were females, and 64.1% were aged younger than 60 years old. Approximately 69.6% of participants had abnormal LDL-C levels; 33.6% had above-optimal levels, 24.3% had borderline high levels, 8.0% had high levels, and 3.7% had very high levels. A total of 17.4% of participants had low high-density lipoprotein cholesterol (HDL-C) and high LDL-C levels, while 14.9% had high triglyceride and LDL-C levels. After controlling for sex, age, religion, education, annual family income, and marital status in the multivariate model, three variables were found to be associated with high LDL-C levels: occupation, the amount of lard used in daily cooking, and glycated hemoglobin (HbA1c). Those who were working as agriculturalists had a 1.34-fold greater chance of having abnormal LDL-C than traders and others (95% CI = 1.09–1.34). Those who used moderate and high quantities of lard in their daily cooking had a 1.45-fold (95% CI = 1.15–1.82) and 1.31-fold (95% CI = 1.04–1.68) greater likelihood of having abnormal LDL-C levels than those who used low quantities, respectively. Those who had abnormal HbA1c levels were less likely to develop abnormal LDL-C levels than those who had normal HbA1c levels (AOR = 0.69, 95% CI = 0.51–92).

**Conclusions:**

Effective public health programs that do not conflict with the cultures of hill tribes are urgently needed, particularly programs encouraging the use of small quantities of lard for daily cooking practices.

**Supplementary Information:**

The online version contains supplementary material available at 10.1186/s12889-021-10577-3.

## Introduction

Low-density lipoprotein cholesterol (LDL-C) is one of the main lipids that directly reflects individual health [1]. The World Health Organization (WHO) reported that elevated LDL-C levels was estimated to result in 2.6 million deaths (4.5% of total) and 29.7 million disability-adjusted life years (DALYs) [1]. Elevated LDL-C levels has been clearly identified as a risk factor for cardiovascular diseases (CVDs) [2]. CVDs are the leading cause of death globally, killing 17.9 million people in 2016 [2]. More seriously, three-fourths of CVD-related deaths were reported in low- and middle-income countries, including Thailand [2], particularly poor economic population [3]. There are several factors that contribute to CVD development, particularly daily dietary patterns, health behaviors [4], and LDL-C levels [5], especially in people living in developing countries [6]. A large budget is required for CVD treatment and care; however, prevention is highly effective if the risk factors in a certain population are clearly known [7].

The United Nations (UN) reported that Thailand had 69 million people with a gross domestic product (GDP) of 455,303 million US$ in 2019 [8], which is one of the upper-middle income countries [9]. The Thailand World Health Organization (WHO) reported that more than 400,000 deaths occurred in Thailand in 2016, and 23.0% of the deaths were due to CVDs [10]. In 2018, the Ministry of Public Health, Thailand reported that 432,943 cases of CVDs were being treated, and 2 CVD-related deaths per hour were reported [11]. The government of Thailand usually allocates a budget of 6.5% of the GDP annually [12] for the care and treatment of CVDs and their complications throughout the health care system, particularly CVDs affecting people who are living in poverty, such as the hill tribe people in northern Thailand.

In the last century, hill tribe people have migrated from South China to Thailand, especially to northern regions such Chiang Rai Province [13]. The WHO reported that approximately 4 million hill tribe people lived in Thailand in 2018 [14]. There are six main groups: Akha, Lahu, Hmong, Yao, Karen, and Lisu. In 2018, a total of 749 hill tribe villages were located in Chiang Rai Province, with a population of approximately 350,000 people [15]. Many hill tribe villages in Thailand are located approximately 500–1500 m above the mean sea level [13]. Therefore, the average environmental temperature is lower than 25 °C in the whole year [13]. Each tribe has its own culture and lifestyle habits, especially in regard to daily cooking practices. Pork is the most favored meat among hill tribe members [16]. Almost all hill tribe members work in the agricultural sector and favor traditional crops to support their family members [16–18]; additionally, a large proportion of the hill tribes in Thailand live below the poverty level [19]. However, there is little biomedical information on elevated LDL-C levels among the hill tribe population in Thailand.

Therefore, this study aimed to estimate the prevalence of and factors associated with elevated LDL-C levels among hill tribe individuals aged 30 years and over.

## Methods

A cross-sectional study was performed to collect data from the participants. The study was conducted in Chiang Rai Province, Thailand. Members of the six main hill tribes, Akah, Lahu, Hmong, Yao, Karen, and Lisu, who lived in Chiang Rai Province composed the study population. In 2018, there were 749 different villages in Chiang Rai Province, Thailand: 316 Lahu villages, 243 Akha villages, 63 Yao villages, 56 Hmong villages, 36 Karen villages, and 35 Lisu villages [15]. Five villages of each tribe were randomly selected for inclusion in the study, for a total of 30 villages. Hill tribe people aged 30 years and over were the target population. However, those who could not provide essential information regarding the study protocols and those who did not follow a 12-h fasting were excluded from the study.

The sample size was calculated according to the standard formula of a cross-sectional study: *n* = [Z_α/2_PQ]/ e^2^, where Z_α/2_ = 1.96, *P* = 0.30 [20], Q = 0.70, and e = 0.05; after adding 10% to account for any error in the study, 2132 participants were needed for the analysis.

A questionnaire and 5 mL blood specimens were used as the research instruments. The questionnaire was developed from a literature review and through discussion with health care professionals who were working in health institutes in hill tribe villages. Finally, there were 4 parts of the final version of the questionnaire. In the first part, 9 questions were used to collect the general information of the participants, such as age, sex, education, occupation, income, etc. In the second part, 10 questions were used to ask about the health behaviors and substance use of the participants, such as exercise, amount of salt used for daily cooking, amount of lard used in daily life, smoking, alcohol use, and methamphetamine use. In the third part, two small surveys were used to assess stress (ST-5) and depression (PHQ-9). In the last part, physical parameters, such as weight, height, and waist circumference, and some laboratory results were obtained (Additional file 1, Questionnaire).

According to the World Health Organization (WHO), low-density lipoprotein cholesterol (LDL-C) levels was classified into two main groups: normal (< 100 mg/dL) and abnormal (≥100 mg/dL) [21]. Abnormal LDL-C levels was classified into four levels: near or above optimal (100–129 mg/dL), borderline high (130–159 mg/dL), high (160–189 mg/dL), and very high (≥190 mg/dL) [21]. Total cholesterol was classified into three levels: desirable (< 200 mg/dL), borderline high (200–239 mg/dL), and high (≥240 mg/dL) [21]. High-density lipoprotein cholesterol (HDL-C) was classified into three groups: low (< 40 mg/dL), normal (40–59 mg/dL), and high (≥60 mg/dL) [21]. Body mass index (BMI) was classified into three groups: underweight (≤18.09 km/m^2^), normal weight (18.10–22.99 km/m^2^), and overweight (≥23.00 km/m^2^) [22].

The questionnaire was validated by the item objective congruence (IOC) method by three external experts who work in the field: one medical doctor, one public health professional, and one epidemiologist. Then, the questionnaire was tested for reliability by testing 20 selected participants who had similar characteristics as the study population in two hill tribe villages in Muang District, Chiang Rai Province, Thailand. In the process of performing the pilot test, the feasibility, understandability and sequence of the questions were tested. After the pilot test, the second test was performed after revising all issues identified in the pilot test.

According to the process of gathering data in the field, access to the villages was granted by the local governments. After the study, all targeted village headmen were contacted and provided with brief information about the study. Village headmen helped to obtain the list of people living in selected villages. All people who met the study criteria made appointments for data and blood specimen collection at least 3 days in advance. On the date of data collection, all participants were provided with all the information of the study by village health volunteers who were fluent in both Thai and local hill tribe languages. All participants were asked to sign the consent form before providing 3-mL blood specimens. Those who did not follow the 12-h fasting guidelines before blood specimen collection were excluded from the study. Physical examination was performed and was followed by completion of the questionnaire. Blood pressure was measured in the seated position twice in each individual by manual procedure with a 10-min gap between. However, in any case that had a large difference between the first and the second time, the third time was assessed with a 5-min gap from the second round. Participants’ blood pressure was assessed by one research team who had 7 years of working experience as a professional nurse. During the completion of the questionnaire, the health volunteers were asked to help with translation in case the participants needed help. The total process lasted for 25 min for each participant. Specimens were kept in a proper box in the field and transferred to the laboratory for analysis on the same day.

All laboratory procedures were performed at the Mae Fah Luang University Medical Laboratory Center. LDL-C and HDL-C levels were assessed by a direct clearance method, while total cholesterol was assessed by the cholesterol oxidase method. Triglycerides were assessed by the glycerol phosphate oxidase peroxidase (GPO-PAP) method. The latex enhanced immunoturbidimetric method was used for HbA1c detection. All lipid profiles and HbA1c assessments were performed by a standard machine, RANDOX^@^, which is certified by the National Glycohemoglobin Standardization Program (NGSP).

Primarily, questionnaires were coded and double entered into an Excel sheet. The data file was then transferred into the R program (R 4.00, 2020) for analysis. Descriptive statistics were used to explain the general characteristics of participants: continuous data (mean and SD) and categorical data (percentages). Pearson correlation was used to assess the correlations between LDL-C levels and other key markers, such as age, systolic blood pressure (SBP), diastolic blood pressure (DBP), triglyceride levels, HDL-C levels, and HbA1C levels. A chi-square test was used to detect the associations between the levels of different kinds of lipids. The t-test was used to detect the mean differences in HDL-C and LDL-C levels between sexes in 6 tribes. Moreover, logistic regression was used to determine the associations between independent variables or exposures and abnormal LDL-C levels (LDL-C ≥ 100 mg/dL) at a significance level of α = 0.05.

## Results

A total of 2552 participants were recruited into the analysis with no people refused to the study; 65.9% were females, 64.1% were younger than 60 years (mean = 54.1, SD = 13.1), and 79.5% were married. Most participants were Buddhist (54.2%), had no education (76.7%), worked as an agriculturist (48.7%), and had an annual family income equal to or less than 50,000 baht. One-fourth (24.3%) were smokers, 23.4% used alcohol, 60.8% did not exercise, 60.7% were overweight according to their BMI, 49.5% were obese according to their waist circumference, and 18.6% had moderate to high levels of stress. A large proportion (90.4%) consumed moderate to high quantities of salt, 90.3% used moderate to high levels of monosodium glutamate, and 81.4% used moderate to high levels of lard for their daily cooking (Table 1).

**Table 1:** Factors associated with LDL-cholesterol in univariable and multivariable analyses

Factors	Total n (%)	LDL-cholesterol		OR	95%CI	***p***-value	AOR	95%CI	***p***-value
		Abnormal n (%)	Normal n (%)						
**Total**	**2552 (100)**	**1778 (69.7)**	**774 (30.3)**	**N/A**	**N/A**	**N/A**			
**Sex**									
Male	870 (34.1)	613 (70.5)	257 (29.5)	1.06	0.89–1.27	0.553			
Female	1682 (65.9)	1165 (69.3)	517 (30.7)	1.00					
**Age** (years)									
30–39	368 (14.4)	260 (70.7)	108 (29.3)	1.00					
40–49	619 (24.3)	423 (68.3)	196 (31.7)	0.45	0.68–1.19	0.446			
50–59	649 (25.4)	455 (70.1)	194 (29.9)	0.86	0.74–1.29	0.855			
60–69	578 (22.6)	402 (69.6)	176 (30.4)	0.72	0.71–1.26	0.718			
70–79	271 (10.6)	188 (69.4)	83 (30.6)	0.73	0.67–1.33	0.727			
≥ 80	67 (2.6)	50 (74.6)	17 (25.4)	0.51	0.67–2.21	0.509			
**Tribe**									
Akha	714 (28.0)	514 (72.0)	200 (28.0)	1.00					
Lahu	391 (15.3)	257 (65.7)	134 (34.3)	0.75	0.57–0.97	0.031*			
Hmong	389 (15.2)	281 (72.2)	108 (27.8)	1.01	0.77–1.33	0.930			
Yao	368 (14.4)	247 (67.1)	121 (32.9)	0.79	0.61–1.04	0.097			
Karen	408 (16.0)	289 (70.8)	119 (29.2)	0.95	0.72–1.24	0.680			
Lisu	282 (11.1)	190 (67.4)	92 (32.6)	0.80	0.60–1.08	0.150			
**Religion**									
Buddhist	1383 (54.2)	966 (69.8)	417 (30.2)	1.00					
Christ and Muslim	1169 (45.8)	812 (69.5)	357 (30.5)	0.98	0.83–1.16	0.832			
**Education**									
No education	1957 (76.7)	1355 (69.2)	602 (30.8)	1.00					
Primary school	365 (14.3)	255 (69.9)	110 (30.1)	1.03	0.81–1.31	0.812			
Secondary school or higher	230 (9.0)	168 (73.0)	62 (27.0)	1.20	0.88–1.64	0.236			
**Occupation**									
Unemployed	628 (24.6)	441 (70.2)	187 (29.8)	1.24	0.98–1.57	0.067	1.26	0.98.1.61	0.070
Agriculturist	1243 (48.7)	891 (71.7)	352 (28.3)	1.33	1.09–1.63	0.005*	1.34	1.09–1.64	0.006*
Trader and other	681 (26.7)	446 (65.5)	235 (34.5)	1.00			1.00		
**Annual income** (baht)									
≤ 50,000	1816 (71.2)	1271 (70.0)	545 (30.0)	1.00					
50,001-100,000	527 (20.7)	358 (67.9)	169 (32.1)	0.91	0.74–1.12	0.366			
≥ 100,001	209 (8.2)	149 (71.3)	60 (28.7)	1.07	0.78–1.46	0.697			
**Marital status**									
Single	138 (5.4)	103 (74.6)	35 (25.4)	1.00					
Married	2039 (79.9)	1415 (69.4)	624 (30.6)	0.77	0.52–1.14	0.196			
Ever married	375 (14.7)	260 (69.3)	115 (30.7)	0.77	0.49–1.19	0.242			
**Smoking**									
No	1932 (75.7)	1350 (69.9)	582 (30.1)	1.00					
Yes	620 (24.3)	428 (69.0)	192 (31.0)	0.96	0.79–1.17	0.691			
**Alcohol use**									
No	1955 (76.6)	1364 (69.8)	591 (30.2)	1.00					
Yes	597 (23.4)	414 (69.3)	183 (30.7)	0.98	0.80–1.20	0.844			
**Exercise**									
No	1551 (60.8)	1078 (69.5)	473 (30.5)	1.12	0.86–1.47	0.407			
Sometimes	719 (28.2)	511 (71.1)	208 (28.9)	1.21	0.90–1.63	0.209			
Regularly	282 (11.1)	189 (67.0)	93 (33.0)	1.00					
**Amount of salt use for cooking**									
Low	244 (9.6)	160 (65.6)	84 (34.4)	1.00					
Moderate	1115 (43.7)	788 (70.7)	327 (29.3)	1.27	0.94–1.70	0.117			
High	1193 (46.7)	830 (69.6)	363 (30.4)	1.20	0.90–1.61	0.219			
**Amount of monosodium glutamate use for cooking**									
Low	248 (9.7)	159 (64.1)	89 (35.9)	1.00					
Moderate	1006 (39.4)	712 (70.8)	294 (29.2)	1.36	1.01–1.82	0.042*			
High	1298 (50.9)	907 (69.9)	391 (30.1)	1.30	0.98–1.73	0.073			
**Amount of lard used for cooking**									
Low	476 (18.7)	304 (63.9)	172 (36.1)	1.00			1.00		
Moderate	1214 (47.6)	872 (71.8)	342 (28.2)	1.44	1.15–1.81	0.001*	1.45	1.15–1.82	0.001*
High	862 (33.8)	602 (69.8)	260 (30.2)	1.31	1.03–1.66	0.025*	1.32	1.04–1.68	0.025*
**Stress** (ST-5)									
Low	2076 (81.3)	1441 (69.4)	635 (30.6)	1.00					
Moderate	399 (15.6)	280 (70.2)	119 (29.8)	1.04	0.82–1.31	0.762			
High	77 (3.0)	57 (74.0)	20 (26.0)	1.26	0.75–2.11	0.388			
**Depression**									
No	2245 (88.0)	1564 (69.7)	681 (30.3)	1.00					
Yes	307 (12.0)	214 (69.7)	93 (30.3)	1.00	0.77–1.30	0.988			
**BMI**									
Normal weight	196 (7.7)	131 (66.8)	65 (33.2)	1.00					
Underweight	807 (31.6)	555 (68.8)	252 (31.2)	0.52	0.38–0.72	< 0.001*			
Overweight	1549 (60.7)	1092 (70.5)	457 (29.5)	1.18	0.86–1.61	0.292			
**Waistline**									
Normal	1290 (50.5)	900 (69.8)	390 (30.2)	1.00					
Obese	1262 (49.5)	878 (69.6)	384 (30.4)	0.99	0.83–1.17	0.915			
**HbA1c** (mg/dl)									
Normal (< 6.5)	2349 (92.0)	1652 (70.3)	697 (29.7)	1.00			1.00		
Abnormal (≥6.5)	203 (8.0)	126 (62.1)	77 (37.9)	0.69	0.51–0.93	0.014*	0.69	0.51–0.92	0.013*

* Binary logistic regression was used to detect the associations at significance level α = 0.05

According to LDL-C levels, 69.6% had abnormal LDL-C; 33.6% had a level from 100 to 129 mg/dL (above optimal), 24.3% had a level from 130 to 159 mg/dL (borderline high), 8.0% had a level from 160 to 189 mg/dL (high), and 3.7% had a level equal to or greater than 190 mg/dL (very high). Additionally, the proportion of abnormal LDL-C according to sex (*p*-value = 0.533) and age categories (*p*-value = 0.908) were not significantly different.

In the univariate model, five factors were found to be associated with high LDL-C levels among the participants: tribe, occupation, amount of monosodium glutamate used for cooking, amount of lard used for daily cooking, BMI, and HbA1C. After controlling for sex, age, religion, education, annual family income, and marital status in the multivariate model, three variables were found to be associated with high LDL-C levels; occupation, the amount of lard use in daily cooking, and HbA1C. Those who were working as agriculturalist had a greater chance of having abnormal LDL-C levels than those traders and other with 1.34 times (95%CI = 1.09–1.34). Those who used moderate and high quantities of lard in their daily cooking practice had a greater chance of having abnormal LDL-C levels than those who used low quantities, with 1.45-times (95% CI = 1.15–1.82) and 1.32-times (95% CI = 1.04–1.68) greater likelihoods, respectively. Those who had abnormal HbA1C were less likely to develop abnormal LDL-C levels than those who had normal HbA1C (AOR = 0.69, 95%CI = 0.51–0.92) (Table 1).

Regarding the correlations between triglycerides and other biomarkers, LDL-C levels were found to be significantly correlated with HbA1c (*r* = 0.039, *p*-value< 0.050), HDL-C (*r* = − 0.244, *p*-value< 0.001), and triglyceride levels (*r* = − 0.099, *p*-value< 0.001). However, when the data were classified by sex, only one variable was found to have a statistically significant correlation with LDL-C levels in males, i.e., triglycerides (*r* = − 0.099, *p*-value< 0.001), and four variables were found to have statistically significant correlations with LDL-C levels in females, i.e., SBP (*r* = 0.049, *p*-value = 0.046), HbA1C levels (*r* = 0.073, *p*-value = 0.003), HDL-C levels (r = 0.237, *p*-value< 0.001), and triglyceride levels (r = − 0.081, *p*-value = 0.001) (Table 2).

**Table 2:** Correlations between LDL and key-biomarkers among the participants

Group	Statistics	Age	SBP	DBP	HbA1C	HDL-C	Triglyceride
**Total**	**r** *(p-value)*	**0.015** *(0.457)*	**0.023** *(0.246)*	**0.023** *(0.242)*	**0.039** (0.050^*^)	**0.244** (< 0.001^*^)	**−0.099** (< 0.001^*^)
**Male**	**r** *(p-value)*	**0.012** *(0.721)*	**−0.027** *(0.421)*	**0.000** *(0.988)*	**−0.032** *(0.073)*	**0.260** *(0.237)*	**−0.136** (< 0.001^*^)
**Female**	**r** *(p-value)*	**0.015** *(0.534)*	**0.049** (0.046^*^)	**0.036** *(0.144)*	**0.073** (0.003^*^)	**0.237** (< 0.001^*^)	**−0.081** (0.001^*^)

*Spearman’s correlation was used to detect the correlations between variables at the significant level α = 0.05; *SBP* Systolic blood pressure; *DBP* Diastolic blood pressure; *HDL-C* High density lipoprotein cholesterol; *LDL-C* Low density lipoprotein cholesterol; HbA1c Glycosylated hemoglobin

When HDL-C and LDL-C levels were assessed in closer detail, it was found that a large proportion (17.4%) had low HDL-C levels (< 40 mg/dL) and elevated or abnormal LDL-C levels (≥100 mg/dL). Another 14.9% had high triglyceride and LDL-C levels. However, there were statistically significant differences between the proportions of the different levels of these lipids among the participants (Table 3). Karen people had the highest mean HDL-C levels (50.08 mg/dL), followed by Yao (49.26 mg/dL) and Lisu people (45.70 mg/dL). Only the Lahu tribe had a significant difference in the mean HDL-C levels between males and females (*p*-value = 0.003). The highest mean LDL-C levels were found in the Karen tribe (122.22 mg/dL), followed by the Hmong (120.15 mg/dL) and Akha (119.96 mg/dL) tribes. And only the Hmong tribe had a significant difference in the mean HDL-C levels between males and females (*p*-value = 0.025) (Table 4).

**Table 3:** Associations LDL-C according to HDL-C, total cholesterol, and triglycerides

Lipids	Levels (mg/dL)	LDL-C (mg/dL)					χ^**2**^ (*p*-value)
		***< 100***	***100–129***	***130–159***	***160–189***	***≥190***	
**HDL-C**	***< 40***	362 (44.9)	278 (34.5)	143 (17.7)	18 (2.2)	5 (0.6)	< 0.001*
	***40–59***	358 (24.1)	472 (28.8)	423 (28.5)	165 (11.1)	66 (4.4)	
	***≥60***	54 (20.6)	107 (40.8)	55 (21.0)	22 (8.4)	24 (9.2)	
**Total cholesterol**	***< 200***	697 (49.0)	563 (39.6)	153 (10.8)	9 (0.6)	0 (0.0)	< 0.001*
	***200–239***	29 (4.0)	264 (36.1)	346 (47.3)	81 (11.1)	11 (1.5)	
	***≥240***	48 (12.0)	30 (7.5)	122 (30.6)	115 (28.8)	84 (21.1)	
**Triglycerides**	***< 150***	427 (28.7)	508 (34.1)	375 (25.2)	116 (7.8)	62 (4.2)	< 0.001*
	***150–199***	80 (19.1)	142 (34.0)	135 (32.3)	46 (11.0)	15 (3.6)	
	***200–499***	267 (41.3)	207 (32.0)	111 (17.2)	43 (6.7)	18 (2.8)	

*Chi-square was used to detect the associations at the significant level α = 0.05

**Table 4:** The HDL-C and LDL-C levels in different tribes

Tribe	Sex	HDL-C		t-test	***p***-value	LDL-C		t-test	***p***-value
		Mean (mg/dL)	SD			Mean (mg/dL)	SD		
**Akha**	Total	44.57	11.24			119.96	34.02		
	Male	44.29	10.47	−0.46	0.646	121.44	33.36	0.79	0.430
	Female	44.71	11.58			119.28	34.33		
**Lahu**	Total	41.16	10.71			115.89	36.51		
	Male	38.98	9.67	−2.97	0.003*	113.56	35.29	−0.92	0.358
	Female	42.33	11.06			117.13	37.15		
**Hmong**	Total	43.53	11.27			120.15	36.23		
	Male	43.55	10.31	0.034	0.973	125.54	37.90	2.25	0.025*
	Female	43.51	11.82			117.02	34.92		
**Yao**	Total	49.26	13.14			118.08	34.64		
	Male	48.49	13.21	−0.74	0.462	119.44	33.12	0.49	0.625
	Female	49.59	13.12			117.50	35.31		
**Karen**	Total	50.08	12.44			122.22	35.84		
	Male	50.01	13.68	−0.09	0.926	121.78	36.00	−0.19	0.852
	Female	50.13	11.72			122.47	35.82		
**Lisu**	Total	45.70	11.19			117.73	36.41		
	Male	44.99	11.88	−0.85	0.398	113.78	38.19	−1.45	0.148
	Female	46.15	10.75			120.22	35.13		

* T-test was used to detect the associations at significance level α = 0.05

## Discussion

The hill tribe people aged 30 years and over in this study had low levels of education and low economic statuses. A large proportion of the participants did not exercise and were overweight, and one-fourth of the participants used substances. Most of the participants favored salty foods and used a large amount of lard for their daily cooking practice. The prevalence of high-to-very-high LDL-C levels was 60.7%. Additionally, daily lard use for cooking practice was determined to be a risk factor for high LDL-C levels among hill tribe people.

The prevalence of abnormal LDL-C levels among hill tribe people aged 30 years and above was high and higher than that among people in Iran [23], which was reported as 40.0%. Al-Hassan et al. [24] also reported that the prevalence of abnormal HDL-C levels was 12.8%. However, a study conducted in northwestern China [25] found that the prevalence of abnormal LDL-C levels was 60.9%, which was close to that in our study. The prevalence of Thai adults was reported to be 29.6% (29.5% in males and 29.5% in females) [20]. Aekplakorn et al. [20] also reported that people who lived in urban areas such as Bangkok (42.2%) had the highest risk of having high LDL-C levels, followed by people who lived in southern, central, and northern Thailand. This reflects that people living in urban areas are more likely to have high LDL-C levels than those who live in rural areas. However, it is very specific to hill tribe people, who reported an overall prevalence of high LDL-C levels of 60.9% among people aged 30 years and over. This may be due to the diet of the hill tribe people and other lifestyle factors associated with the Chinese culture since they migrated from China and still have many practices based on their original culture. Moreover, the classification of LDL-C is important to classify the outcome of a study. Different guidelines from different institutes suggest different patterns of LDL-C classification.

The finding in our study was presented that some category of occupation had a greater chance of having abnormal LDL-C levels than another category; those who worked in agricultural sector and unemployed group had a greater chance of having abnormal LDL-C levels than those traders. While looking closer in the income among three occupations, it was found that 92.7% of unemployed group had their annual income ≤100,000 baht, 88.7% of agriculturist had their annual income ≤100,000 baht, while only 66.6% of traders had their annual income ≤100,000 baht. This is supported by a study conducted in Chili which presented that lower income led to have high stress, and accelerated abnormal of LDL-C levels eventually [26]. Moreover, this finding was consisted with a study conducted in Thailand, which reported that working as agriculturalist contributed in the abnormal of LDL-C levels significantly [27]. A study in North American and Europe was clearly presented on the association between some categories of occupation and abnormal LDL-C levels especially those who were working under high stress and low paid such as agriculturists [28]. Another study in remote areas in Australia, it was found that those who were agricultural workers had a greater chance of having abnormal LDL-C levels than other groups significantly [29].

In our study, it was found that the quantity of lard use was associated with abnormal LDL-C levels among hill tribe people aged 30 years and over. Normally, the hill tribe people use animal oil for cooking (lard) [13], and use a large amount of lard in their practice which is similar to Chinese cooking style. This specific cooking practice is supported by their origin in China before moving down to Thailand a long a couple centuries [13, 16]. In China, the environmental temperature is average in low, then having high volume of cooking oil in daily life is common to maintain body temperature. However, in their current living environment in Thailand where are a tropical zone, the environmental temperature is higher than in China but the hill tribe people still eating high volume of cooking oil. Then, this could lead overweight (60.7%) and having abnormal LDL-C levels. Very interestingly, after seeking other studies in both Thai and English from different sources of medical and public health sciences databased, there is no scientific information relevant to quantity of lard and LDL-C levels available.

In our study, it was also found that LDL-C levels had a positive correlation with HbA1C and a negative correlation with HDL-C and triglycerides. A closer examination revealed that LDL-C levels had a negative correlation only with triglycerides and had positive correlations with SBP, HbA1C, HDL-C levels, and triglycerides in females. This was supported by a study conducted by Bhattacharjee et al. [30] that reported that LDL-C levels was significantly correlated with HbA1C in a positive direction among myocardial infarction patients. A study among diabetes patients in Iran found that LDL-C levels was significantly positively correlated with both DBP and SBP [31]. Another study in Poland [32] showed that LDL-C levels and triglycerides levels had a positive correlation with triglycerides in both male and female participants. A study in the United States [33] and a study in Japan [34] also clearly demonstrated the positive correlation of LDL-C levels with both DBP and SBP. This may be most the hill tribe people consume a large amount of lard in daily life but a few proportions have regular exercise (11.1%). Moreover, according to the BMI, 60.7% were classified as overweight which is a good indicator that the hill tribe people have high cooking oil dietary and low physical activity problems.

A few limitations were found in this study. The language barrier was the major problem in the study; however, the language barrier was addressed by the village heath volunteers who were trained for at least 1 h to understand the whole content of the questionnaire before starting the interview. Second, some significant variables in the questionnaire were not clearly categorized before assessment in the field, such as the type and frequency of smoking and the frequency and volume of drinking alcohol, which could impact the interpretations of the findings. In addition, some measures assessing the volume of lard, salts, and monosodium glutamate used are difficult to apply for the hill tribe context, which has various cooking practices, including different sizes of utensils used and different family members having food each time. The accuracy of measurements in terms of health behaviors and cooking practices are of great concern to all studies while working in hill tribe people. In addition, the interpretation of the correlations between LDL-C and key biomarkers can be more informative by accounting for total calories and total saturated fat intake, visceral obesity and insulin resistance.

## Conclusion

The hill tribe people in Thailand are working in the agricultural sector and often live with poor economic status and illiteracy. They do not exercise in daily life and favor salty food and heavy use lard for cooking practice. Moreover, a large proportion of these individuals are overweight with high LDL-C levels. These individuals are at high risk of developing cardiovascular diseases that could lead to several complications and eventually require health care services. Therefore, effective public health interventions should be implemented for the population to improve personal health behaviors and to reduce the quantity of lard and salt used in daily cooking practices. Additionally, these individuals should be educated on healthy behaviors, a healthy diet and exercise to reduce the likelihood of having health problems related to high LDL-C levels.

## Supplementary Information

**Additional file 1.** Questionnaire

## Data Availability

The datasets used in the study are available from the corresponding author on reasonable request.

## Abbreviations

BMI: Body mass index
CI: Confident interval
CVD: Cardiovascular diseases
DALYs: Disability-adjusted life years
GDP: Gross domestic product
DBP: Diastolic blood pressure
HbA1C: Glycated hemoglobin
HDL-C: High-density lipoprotein cholesterol
IOC: Item objective congruence
LDL-C: Low-density lipoprotein cholesterol
OR: Odds ratio
SBP: Systolic blood pressure
SD: Standard deviation
UN: United Nations
WHO: World health Organization
