# Supplementary material for: Factors associated with elevated low-density lipoprotein cholesterol levels among hill tribe people aged 30 years and over in Thailand: a cross-sectional study
Source: BMC Public Health. 2021 Mar 12;21:498. doi: 10.1186/s12889-021-10577-3 (PMC7953743; doi:10.1186/s12889-021-10577-3)
Supplement: Supplementary file 1 — Additional file 1. Questionnaire [file 12889_2021_10577_MOESM1_ESM.pdf]

Questionnaire

Part I general information

1. Sex

Male Female

2. Age……………………Year
3. Education
   - No-education
   - Primary school
   - High school
   - University degree
4. Tribe
   - Akha
   - Lahu
   - Hmong
   - Yao
   - Karen
   - Lisu
5. Religion
   - Buddhist
   - Christian
   - Other
6. Occupation
   - Unemployed
   - Agriculturalist
   - Trader or other
7. Annual income…………………….baht
8. Marital status
   - Single
   - Married
   - Ever married
9. Having debt
   - Yes
   - No

Part II Health behaviors and substance use

1. Exercise
   - No
   - Sometimes
   - Regular
2. Amount of salt use for cooking
   - Low
   - Moderate
   - High
3. Amount of monosodium glutamate use for cooking
   - Low
   - Moderate
   - High
4. Amount of cooking oil used for cooking
   - Low

Moderate
High

5.Alcohol use
Yes
No
6. Smoking
Yes
No
7. Amphetamine use
Yes
No
8. Opium use
Yes
No
9. Glue use
Yes
No
10. Marijuana use
Yes
No

Part III ST-5 and PHQ-9

ST-5 (Stress screening question)
1. Have you had sleep problem or over sleeping within 2 week prior? 0 1 2 3
2. Have you had concentration problem within 2 week prior?? 0 1 2 3
3. Have you had anxious within 2 weeks prior? 0 1 2 3
4. Have you had boring feeling within 2 weeks prior? 0 1 2 3
5. have you had isolation from people within two weeks prior? 0 1 2 3

PH-9
1. Little interest or pleasure in doing things 0 1 2 3
2. Feeling down, depressed, or hopeless 0 1 2 3
3. Trouble falling or staying asleep, or sleeping to much 0 1 2 3
4. Feeling tired or having little energy 0 1 2 3
5. Poor appetite or overrating 0 1 2 3
6. Feeling bad about yourself 0 1 2 3
7. Trouble concentrating about things 0 1 2 3

8. Having slowly moving or speaking 0 1 2 3

9. Thoughts that you would be better off dead. 0 1 2 3

Part IV Physical examination

Weight…………………………………………….....kg
Height………………………………………..………cm
Blood pressure (1)……………..mmHg, (2)………………mmHg, (3)……………mmHg
Waist circumference ……………………………..cm
LDL ………………………………………………... mg/dL
HDL………………………………………………...mg/DL
Hba1c
